# Protective Efficacy of the Calicivirus Valency of the Leucofeligen Vaccine against a Virulent Heterologous Challenge in Kittens

**DOI:** 10.1155/2013/232397

**Published:** 2013-06-20

**Authors:** Cynthia Lesbros, Virginie Martin, Wojciech Najbar, Annaele Sanquer, David Mcgahie, Hyone-Myong Eun, Sylvie Gueguen

**Affiliations:** ^1^R&D Department, Virbac, 13éme rue, LID, BP 27, 06511 Carros Cedex, France; ^2^Medical Department, Virbac, 13éme rue, LID, BP 27, 06511 Carros Cedex, France

## Abstract

Feline calicivirus (FCV) is a common feline pathogen with a potential for antigenic diversity. This study aimed to evaluate and characterize the protective efficacy of the FCV-F9 valency of a tetravalent vaccine, Leucofeligen, against challenge with an unrelated strain. Ten 9-week-old kittens were vaccinated while 10 remained as unvaccinated controls. The vaccinated cats received Leucofeligen twice subcutaneously with a 3-week interval. Four weeks after the second vaccination, all cats were challenged with virulent heterologous FCV and followed up for 21 days, monitoring their general condition, clinical signs, and immunological responses. During the vaccination phase, rectal temperatures and body weights were indistinguishable between the two groups. Only vaccinated cats showed FCV-specific seroconversion (both total and neutralizing antibodies). In the first week after challenge, the vaccinated cats had an 82.6% reduction in median clinical score compared to controls. Leucofeligen was thus shown to provide a significant clinical protection to kittens challenged with heterologous virulent FCV. This protection was similar whether the cats had neutralizing antibody or not, indicating a key role for cellular immunity in the overall protection. This also suggests that previously reported seroneutralisation studies may underestimate the level of cross-protection against field strains obtained with this modified live FCV-F9 vaccine.

## 1. Introduction

Feline calicivirus (FCV) is a common pathogen of cats normally infecting the oral cavity and upper respiratory tract. The initial infection generally results in acute clinical signs such as fever and lingual or oral ulceration, in addition to sneezing, rhinitis, and conjunctivitis [1]. However, cats infected with FCV may not exhibit overt clinical disease when they are persistently infected or when they are infected with FCV isolates that are only mildly pathogenic. Other nontypical FCV infections have also been observed that produce various clinical symptoms such as lameness or diarrhea, and hypervirulent strains causing virulent systemic disease (or VSD) are diagnosed sporadically [2, 3].

FCV has a single-stranded positive-sense RNA genome of ~7.7 kb. The replication of FCV, like other RNA viruses in general, results in a high proportion of genomic as well as antigenic variants. Indeed the overall identity of FCV isolates collected worldwide was reported to be approximately 80% for the variable and immunodominant regions C to E of the capsid gene [4, 5]. This genomic diversity gives rise to antigenic variants and argues for the importance of cross-reactive vaccines that can provide protection against antigenically distinct FCV strains. 

A tetravalent vaccine, Leucofeligen, was developed in our laboratory containing live attenuated FCV, feline herpesvirus (FHV-1, also known as feline rhinotracheitis virus) and feline panleukopenia virus (FPV), as well as the recombinant p45 antigen of feline leukaemia virus (FeLV).

When developing this vaccine, we decided to evaluate the protective efficacy of the FCV valency against an unrelated virulent heterologous challenge strain and also to attempt to characterize the nature of the protective immune response.

## 2. Materials and Methods

The study was carried out in accordance with the Good Laboratory Practice guidelines, and additionally in accordance with the recommendations issued in the European Pharmacopoeia [6].

### 2.1. Animals and Study Protocols

Twenty specific-pathogen-free (SPF) European kittens, 9 weeks old, were randomly assigned to 2 groups: control (unvaccinated, hereafter designated group C) and vaccinated (hereafter designated group V). Cats were acclimatized for 6 days to the animal housing conditions (12 h light/dark cycle, 18 ± 3°C, 55 ± 10% humidity, with free access to water). Each group was housed in a separate airspace in the animal housing facility.

Group V cats were vaccinated twice at a 3-week interval (day 0 and day 21) by subcutaneous injection (1 mL) according to the recommendations of the manufacturer. In order to better assess the local tolerance of the injections, the first injection was given half way between the shoulder and hip on the right side, and left side was used for the second injection. Group C cats did not receive any injections.

Four weeks after the second vaccination, on day 49, equivalent to postchallenge time 0 (= pct0), all cats were challenged with a virulent heterologous strain of calicivirus (FCV-255). Cats were first anesthetized and then inoculated intranasally with 10^7.5^  TCID_50_/cat of FCV-255 suspension using a volume of 0.25 mL/nostril.

### 2.2. Test Vaccine

Leucofeligen was granted a pan-European marketing authorization (centralised procedure) in 2009. It is presented as a freeze-dried fraction containing the live attenuated viruses, that is, FCV (F9), FHV-1 (F2), and FPV (LR72), and a liquid fraction containing the recombinant FeLV-envelope antigen p45 (derived from the gp70 of FeLV) with aluminium hydroxide and QA-21 adjuvants. The calici valency in the freeze-dried fraction was formulated at the minimum accepted titre for this vaccine. The vaccine vials were stored at +5°C and were reconstituted immediately prior to use by rehydrating the freeze-dried fraction with the liquid fraction.

### 2.3. Monitoring

 In the vaccination phase, cats were monitored daily for general health status (food intake, appearance of feces, and behaviour/depression). The animals were weighed weekly. In the postchallenge phase, clinical examinations were performed daily, and the clinical status was evaluated according to a scoring system based on that specified in the pharmacopoeia monograph [6] (see Table 1). For comparison between groups, two methods were used: the maximum clinical score (taking the highest recorded score for each parameter in each cat) was used to assess differences in the severity of the clinical picture, and the cumulative score (adding the scores recorded each day for each parameter in each cat) was used to evaluate the impact of the duration of each sign. For both of these comparisons, scores for rectal temperature, ulceration, nasal discharge, ocular discharge, and weight loss were included. As weight loss represents a single score on day pct7 (representing the loss over the previous week) it has the same impact on both maximum and cumulative scores.

Blood samples were collected from the animals in uncoated tubes for serological assessment on days 0, 21, 35, 49 (= pct0), 56 (= pct7), 63 (= pct14), and 70 (= pct21).

### 2.4. Serological Assessments

The serological assessments involved assaying IgG anticalicivirus antibody (Ab) and anti-calicivirus neutralizing antibody (NAb) titres. 

#### 2.4.1. IgG Ab

 Titres of IgG against calicivirus were assessed using an immunofluorescent antibody assay. Briefly, 50 *µ*L of two-fold dilutions of each serum (from 1/64 to 1/8192) was added to a 96-well plate containing acetone-fixed CRFK cells infected with FCV-F9. 50 *µ*L of a positive serum and 50 *µ*L of a negative serum were diluted in the same way and used as controls. They were incubated for 1 hour at 37°C and revealed with a fluorescein-conjugated antifeline IgG antibody and a solution of Evans Blue. The positivity threshold was 1/128.

#### 2.4.2. Neutralising Ab

Titres of NAb were determined to the homologous FCV-F9. Briefly, 50 *µ*L of each serum was diluted with L15/McCoy's medium to provide 6 2-fold dilution steps between 1/8 and 1/256. 200 *µ*L per dilution was incubated for 1 hour with 200 *µ*L of FCV-F9 suspension at a concentration of approximately 100 TCID_50_ to allow viral neutralisation. 50 *µ*L of each mixture was then added to 6 plates of a 96-well plate containing 70% confluent CRFK cells. After 6 days of incubation, the characteristic cytopathic effect was assessed. The titre was determined by the Spearman and Karber method [7] and considered as negative when inferior to 0.9 which was the detection threshold.

### 2.5. Statistical Tests

All statistical analyses were performed using the S-PLUS 6.2 software package (Insightful, Paris). For the comparison of rectal temperatures, body weights, and clinical scores between group C and group V, Student's *t*-tests, one-sided Wilcoxon rank sum tests, and/or the one-sided Dunnett method were applied. A *P* value <0.05 was considered as significant.

### 2.6. Ethical Approval

This work was performed under the supervision of the Ethical Committee of Virbac, and in accordance with the requirements of the official European Pharmacopoeia [6].

## 3. Results

### 3.1. Vaccinal Phase

#### 3.1.1. General Health Parameters

During the vaccination phase (day 0 to 49), all cats remained in normal health with a steady increase in their body weights and with normal body (rectal) temperatures. No abnormal general or local reactions were noted in relation to the vaccinations administered.

#### 3.1.2. Clinical Signs

No cat in either group displayed any clinical signs during the vaccination phase.

#### 3.1.3. Immune Responses

**Figure 1:**
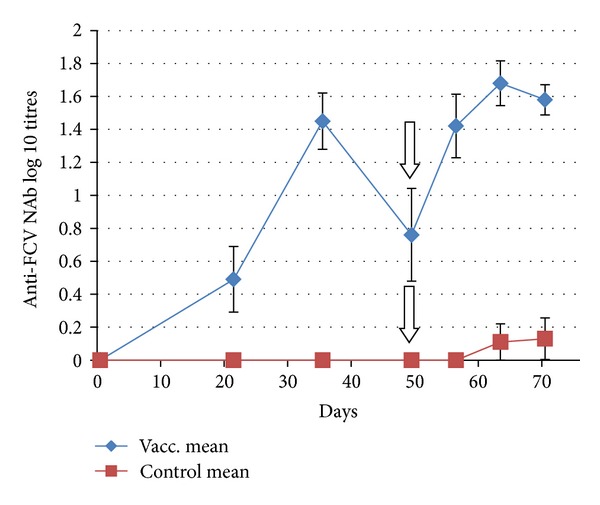
Time courses of homologous anti-FCVF9-NAb responses in cats after vaccination with FCV-F9 (group mean, *n* = 10). Error bars denote the SEM. Vertical arrow indicates the day (d49 or pct0) when the cats were challenged with virulent heterologous FCV-255.

**Figure 2:**
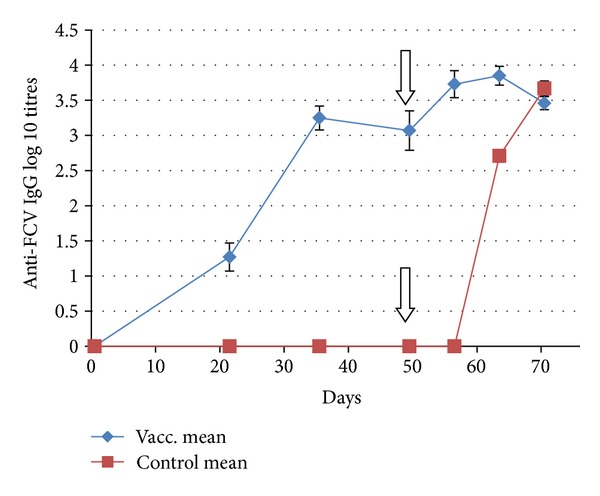
Time courses of anti-FCV-F9-IgG responses in cats after vaccination with FCV-F9 (group mean, *n* = 10). Error bars denote the SEM. Vertical arrow indicates the day (d49 or pct0) when the cats were challenged with virulent heterologous FCV-255.

The time course of the Ab responses induced by FCV-F9 vaccination (day 0–49, vaccination phase) is shown in Figure 1 (anti-FCV-NAb) and Figure 2 (anti-FCV-IgG antibodies). Regarding anti-FCV-NAb responses (Figure 1), all vaccinated cats seroconverted by day 35. On day 49 (pct = 0), however, the 5 cats with titres less than or equal to 10^1.3^ had returned to be Nab negative. All control cats remained strictly Nab negative in the same period.

In contrast to the NAb responses, anti-FCV-IgG responses were elicited in a uniform and strong manner in all vaccinated cats by day 35 and they remained high on day 49 (Figure 2).

### 3.2. Postchallenge Phase

#### 3.2.1. General Health Parameters

**Figure 3:**
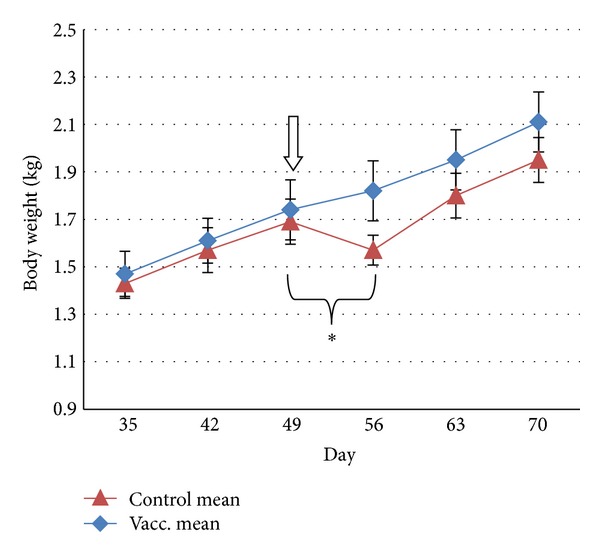
Time points of the body weights (group mean, *n* = 10) before and after challenge with virulent heterologous FCV on day 49 (denoted by down arrow). From day 0 to day 49 (vaccination phase), body weights of the cats were indistinguishable between the two groups. Vertical error bars denote the SEM. The bracketed time period indicates a significant difference in mean weight gain or loss between groups (*P < 0.0001).

At the start of the challenge phase (pct0), there was no difference in the body weights of the two groups (*P* = 1.00). In the first week of the postchallenge phase (pct0 to pct7), the body weights of 9 out of 10 of the group C cats decreased (mean weight loss of 6.75%), in contrast to the group V cats which maintained a normal pattern of weight gain (mean weight gain 4.66%) during this period (Figure 3). The difference in percentage of weight gain or loss during the first week after challenge between the groups was highly significant (*P* < 0.0001). 

**Figure 4:**
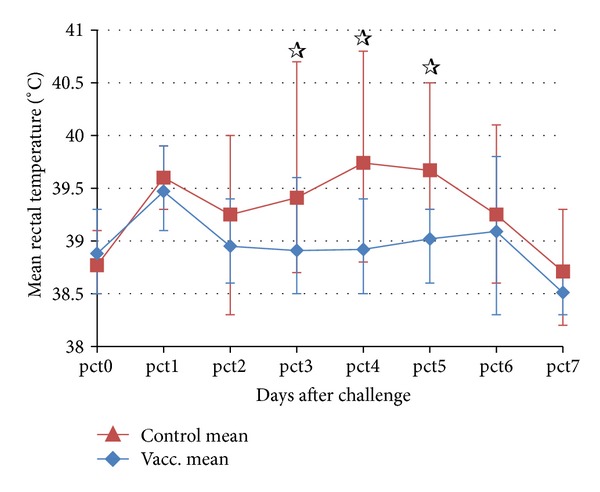
Daily rectal temperatures of the cats (group mean, *n* = 10) after the challenge with heterologous FCV. Error bars denote the maximum and minimum temperatures. Significant differences (*P* < 0.05) between the two groups were observed on days 3, 4, and 5 (*☆*).

During the same time period, hyperthermia (>39.4°C) was observed for every cat in group C on at least 2 of the days, with 4 of the 10 cats presenting peaks of at least 40°C and one of them sustaining a rectal temperature of >40°C for 4 days. In contrast, in group V transient peaks, lasting no more than 1 day, were observed in 7 of the 10 cats. 3 of those were only 39.5°C, and none of the group V cats reached 40°C. Mean rectal temperatures were significantly different between the two groups on days 3 to 5 after challenge (*P* between 0.0002 and 0.0137) and are displayed in Figure 4.

#### 3.2.2. Clinical Signs and Comparison of Scores

During the first week after FCV challenge (pct0 to pct7) a significant difference was observed between the groups regarding the development of clinical signs. 

Nine of the 10 group C cats developed oral and/or nasal ulcers: 4 cats had the maximum score of 3 (corresponding to large and/or numerous ulcers—see Table 1 for details) with one maintaining this maximum score for 12 days, and the other 5 cats had a score of 1 (corresponding to small ulcers, few in number). In contrast, only one cat in group V developed oral ulceration with a score of 3 (for only 4 days), while 4 others developed mild ulceration with a score of 1. Ocular discharge was observed in one group C cat but never in the vaccinated cats. Nasal discharge was far more common, affecting 5 group C cats, but again no group V cats demonstrated nasal discharge at any time. 

**Figure 5:**
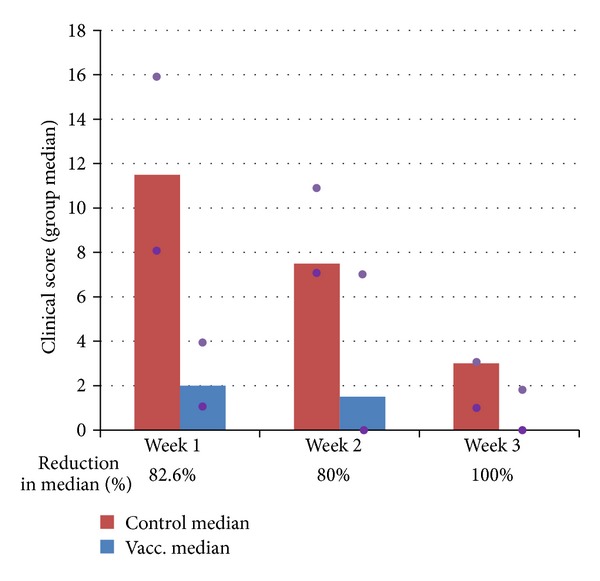
Cumulative clinical scores (weekly median, *n* = 10) of the two groups of cats after heterologous FCV challenge, where Week 1 = pct0 to pct7, Week 2 = pct8 to pct14, and Week 3 = pct15 to pct21. Purple dots indicate the 25% and 75% quartiles.

In general, group V cats developed substantially reduced clinical signs and for a substantially shorter duration compared with the control cats (Figure 5). The maximum clinical scores during the first week after challenge were significantly reduced in group V compared to group C: reduction in the median was 80% (*P* = 0.0002). The reduction in the median of the cumulative clinical scores was also significant at 82.6% (*P* = 0.0004) (Table 2).

After the first week following challenge, and entirely as expected, most cats improved rapidly. However, cumulative scores for ulceration remained significantly different between pct8 and pct21 (*P* = 0.014) with a 95.0% reduction in the vaccinated group during this period.

#### 3.2.3. Immune Responses

Following the heterologous FCV-challenge on day 49 (= pct0), the mean homologous NAb titres (Figure 1) rose substantially (6.6-fold) in the vaccinated group. One cat in this group, which had a nondetectable NAb titre at the time of challenge, had a delayed and only moderate appearance of the NAb titre which could be detected only after pct14. However, the only sign noted for this cat throughout the entire challenge period was a very slight rise in rectal temperature of 0.1°C above the threshold on one single occasion only. The other 4 cats with nondetectable NAb titres at the time of challenge had reseroconverted on pct7. Control cats remained Nab negative throughout the challenge period except for one cat that elicited a detectable level of NAb on pct14 and pct21.

The stimulation of anti-FCV-IgG responses (Figure 2) in the group V cats was rapid and substantial, with a 5-fold increase in mean titres during the first week after the challenge. The anti-FCV-IgG response to challenge in the group C cats began later but was then rapid and intense. From pct14, all 10 cats developed substantial titres of anti-FCV-IgG in a very uniform fashion, in contrast with the virtual absence of NAb. By pct21, the anti-FCV-IgG titres reached levels similar to or even higher than those of the vaccinated cats. 

## 4. Discussion

Due to the variable nature of FCV, the ability to cross-protect against unrelated strains is a key requirement for an efficacious vaccine [1]. The aim of the study was to evaluate and characterize the protective efficacy of a recently licensed combination vaccine, Leucofeligen, against heterologous virulent challenge. During this study all group V cats also seroconverted (produced NAb) to the other live valencies (FHV and FPV) after vaccination, in contrast to the group C cats, none of which seroconverted to FHV or FPV (data not shown). The protective efficacy of Leucofeligen with respect to the FeLV valency, which was previously evaluated and reported elsewhere [8], was not assessed during this study. 

The choice of FCV-255 as a heterologous challenge strain was legitimate due to the dissimilarity of the two virus strains. It seems that the maximum dissimilarity detected within the calicivirus pool is in the order of 30% [5], and one study demonstrated that FCV-255 and F9 have only 70% homology within region E of the capsid protein, which is a similar level of relatedness to that seen with other highly diverse strains including some of the highly virulent strains examined in that study [9]. In one seroneutralisation study performed with sera raised to FCV-255 and F9, it was clear that amongst the field strains neutralised by the highest antibody dilution titres of one of the sera there was rarely a corresponding high titre for the other serum [10].

The level of clinical protection achieved in this study was very encouraging. Current guidelines [11] suggest that in high-risk environments, such as rescue shelters, we can reasonably expect vaccines for feline calicivirus to provide around 60 to 70% protection due to the higher levels of exposure. In this study exposure was guaranteed at high doses by the direct intranasal administration of the pathogenic virus. Therefore achieving an 82.6% reduction in median scores over the first week after challenge in such circumstances is an excellent result. As the disease is self-limiting in immunocompetent cats, and most cats begin to heal spontaneously in around 7 to 10 days after the onset of the disease, the first week after challenge is the most appropriate period to assess the benefit obtained as a result of vaccination.

The signs which are most visible for the pet owner (nasal discharge, ocular discharge, and weight loss) were completely prevented during this time. Likewise severe fever likely to result in noticeable lethargy and malaise was also completely prevented. In this context, it can be noted that, in the monograph for FHV, severe hyperthermia (40°C or higher) is given a higher score, allowing the severity of this sign to be reflected in the final scores [12]. We remained within the FCV monograph for the scoring used in this study, but had the modified score used in the recent FHV monograph been used, the difference between groups would have been even greater due to the fact that the vaccine completely prevented severe hyperthermia. 

Ulcers are noticeable to the owner only when widespread and severe. Therefore the reduction in both the duration and the severity of the ulceration also means that it is unlikely that an owner would be aware of these ulcers in a vaccinated cat in the great majority of cases. Complete prevention of ulceration is not expected in such a study with direct administration of high doses of pathogenic virus. Indeed we can assume that the levels of mucosal IgA required to block such doses of virus are unlikely to be achievable, meaning that some cytopathic effect is inevitable. Nevertheless, a reduction in the severity and duration of ulceration and fever is probably the two parameters which most benefit the welfare of the animal, one of the main reasons to use FCV vaccines.

Following the serological responses in addition to the clinical responses was also very interesting in this study. IgG antibodies levels rose to high titres during the challenge phase in all cats in this study, regardless of the severity of the clinical signs, and therefore appear to be simply a marker of exposure to the virus and do not indicate useful information about the level of protection achieved.

During the vaccination phase (day 0–49) of the present study, the titres of anti-FCV-NAb produced by the vaccinated cats were neither high nor long lasting. This is very much in line with previous published work, where studies based on seroneutralisation required use of altered and intense protocols of exposure to the vaccine strains to induce sufficiently high titres to permit cross-neutralisation assays to be performed [9, 10, 13–15]. This raises the possibility that some of the neutralising responses in these studies could also be due to other possibly nonspecific, immune mechanisms not seen in normally vaccinated cats [10].

At the time of the heterologous challenge, cats in our study with detectable levels of NAb were protected against severe disease. However, by contrast, half the vaccinated cats had no detectable NAb, and the lack of NAb was not correlated with susceptibility to the infection.

As a result, we can conclude that high NAb titres appear to function as a marker that an active immune response has been produced but are probably not the key protective agent against this virus. The lack of NAb titres in healed (and presumably therefore immune) control cats strongly supports this conclusion.

Such a result is perhaps not entirely surprising. Indeed in terms of antibody protection, circulating antibody has a minimal benefit to offer in terms of protection against a mucosal virus, where perhaps levels of IgA mucosal antibody would be more interesting if it was possible to assay these accurately. More importantly, when using modified live vaccines, it is widely accepted that cell-mediated immunity is likely to be a major factor in the protection induced by the vaccine [16].

In a study focussed on a bivalent inactivated vaccine [9], the authors found that there was no correlation between neutralising antibody titres and clinical protection, although high antibody titres were predictive of clinical protection. However, when a blocking ELISA assay was used, they found that, in cases where there were significant titres to both of the inactivated FCV strains present in the vaccine, there was an 89% predictive value of a reduction in the clinical score of at least 50%. Use of an inactivated vaccine is less likely to induce strong cell-mediated responses. Taken together, our results and those of Poulet et al. [9] may suggest that although antibody levels could be predictive of cross-protection when inactivated vaccines are used, they greatly underestimate cross-protection when modified live F9 vaccines are used, probably due to a stronger role of the cell-mediated immune responses.

This finding on the relevance of NAb titres also confirms the need to perform confirmatory challenge studies in order to be able to draw useful conclusions on the efficacy of an FCV vaccine. It would also be interesting to look further at specific cell-mediated immunity parameters in future studies.

## 5. Conclusion

Our study demonstrated that the combination vaccine, Leucofeligen, could provide a protective efficacy (reduction of clinical score) of 82.6% during the first week after challenge in vaccinated kittens challenged with virulent heterologous FCV. The signs most visible for the pet owner (nasal discharge, ocular discharge, and weight loss) were completely prevented during this time, as was severe fever likely to result in noticeable lethargy or malaise. High titres of neutralising antibody seem to indicate protection, but the absence of NAb does not indicate susceptibility, indicating a role for cell-mediated immunity with this vaccine. It therefore appears that, when modified live F9 vaccines are used, *in vitro* seroneutralisation studies are likely to underestimate the level of cross-protection that may be achieved against field strains *in vivo*.

## Figures and Tables

**Table 1:** Scoring system for the parameters concerning general health conditions and clinical signs.

Symptoms	Description	Notation
Rectal temperature (°C)	37.1–39.4 ≥39.5 ≤37.0	0 1 2

Body weight*	Gain or loss of <3% Loss of ≥3%	0 2

Ulcers (oral and/or nasal)	Absence Small and few Large or numerous	0 1 3

Nasal discharge	Absence Slight Copious	0 1 2

Ocular discharge	Absence Presence	0 1

*Weight loss (%) = 100 × ([pct7] − [pct0])/[pct0].

**Table 2:** Cumulative clinical scores (CS) using the scoring system as defined in Table 1 for the individual cats during the first week after challenge.

Cat ID group V	Cumulative CS Week 1 (pct0 to pct7)	NAb at challenge	Cat ID group C	Cumulative CS Week 1 (pct0 to pct7)	NAb at challenge
1	12	(−)	1	10	(−)
2	3	(−)	2	11	(−)
3	0	+	3	13	(−)
4	1	+	4	8	(−)
5	1	(−)	5	8	(−)
6	4	(−)	6	29	(−)
7	1	+	7	12	(−)
8	4	+	8	7	(−)
9	6	+	9	16	(−)
10	1	(−)	10	18	(−)

Median	2.0		Median	11.5	

Q25%; Q75%	1.0; 4.0		Q25%; Q75%	8.0; 16.0	

Mean [95% CI]	3.3 [0.7; 5.9]		Mean [95% CI]	13.2 [8.5; 17.9]	
